# Aggregation-Induced Red Emission Nanoparticle-Based Lateral Flow Immunoassay for Highly Sensitive Detection of Staphylococcal Enterotoxin A

**DOI:** 10.3390/toxins15020113

**Published:** 2023-01-29

**Authors:** Hanpeng Xiong, Ping Chen, Xirui Chen, Xuanang Shen, Xiaolin Huang, Yonghua Xiong, Yu Su

**Affiliations:** 1 State Key Laboratory of Food Science and Technology, Nanchang University, Nanchang 330047, China; 2 Jiangxi-OAI Joint Research Institute, Nanchang University, Nanchang 330047, China; 3 State Key Laboratory of Food Science and Technology, School of Food Science and Technology, Nanchang University, Nanchang 330047, China

**Keywords:** staphylococcal enterotoxin A, aggregation-induced emission, boronate affinity reaction, lateral flow immunoassay

## Abstract

Staphylococcal enterotoxin A (SEA) has presented enormous difficulties in dairy food safety and the sensitive detection of SEA provides opportunities for effective food safety controls and staphylococcal food poisoning tracebacks. Herein, a novel aggregation-induced emission (AIE)-based sandwich lateral flow immunoassay (LFIA) was introduced to detect SEA by using red-emissive AIE nanoparticles (AIENPs) as the fluorescent nanoprobe. The nanoprobe was constructed by directly immobilising antibodies on boronate-tagged AIENPs (PBA-AIENPs) via a boronate affinity reaction, which exhibited a high SEA-specific affinity and remarkable fluorescent performance. Under optimal conditions, the ultrasensitive detection of SEA in pasteurised milk was achieved within 20 min with a limit of detection of 0.04 ng mL^−1^. The average recoveries of the PBA-AIENP-LFIA ranged from 91.3% to 117.6% and the coefficient of variation was below 15%. It was also demonstrated that the PBA-AIENP-LFIA had an excellent selectivity against other SE serotypes. Taking advantage of the excellent sensitivity of this approach, real chicken and salad samples were further analysed, with a high versatility and accuracy. The proposed PBA-AIENP-LFIA platform shows promise as a potent tool for the identification of additional compounds in food samples as well as an ideal test method for on-site detections.

## 1. Introduction

*Staphylococcus aureus* (*S. aureus*) is a common foodborne pathogen that exists widely in nature. An *S. aureus* count of >10^5^ CFU g^−1^ could cause staphylococcal food poisoning (SFP) by producing staphylococcal enterotoxins (SEs), a class of proteins or peptides that are water soluble and have molecular weights between 26 and 30 kDa [1]. SEs possess an extremely high stability against heat and proteolytic enzymes and display super antigenic activity [2]. Accordingly, SEs maintain their activity and pathogenicity even after strict treatments. An intake of a few micrograms could induce severe toxicity symptoms such as celialgia, vomiting, convulsions and diarrhoea [3]. The main SE serotypes include SEA, SEB, SEC, SED and SEE. Amongst them, SEA has the highest virulence and is the most common type involved in staphylococcal food poisoning. Therefore, the fast and sensitive detection of SEA in dairy products is vital for food safety controls and could provide an effective traceback of SFP outbreaks.

The common analytical technologies for SEA detection include polymerase chain reaction-based methods [4,5], chromatography [6], mass spectrometry [7,8] and ELISAs [9]. Compared with these methods, a lateral flow immunoassay (LFIA) provides advantages of speed, simplicity, field screening, naked-eye identification, a low cost and ease of use, which are much favoured in the fields of biomarker analysis, environmental monitoring and food safety detection [10,11,12]. However, a traditional LFIA that uses gold nanoparticles (AuNPs) as the signal label suffers from a poor visual recognition and low sensitivity owing to the low colorimetric signal intensity of AuNPs [13,14]. As an alternative to a light absorber, fluorophores endow the LFIA with a higher sensitivity owing to their superior contrast and optical signal [15,16]. In addition, compared with a single fluorophore label, various fluorescent nanoparticles loaded with fluorescent dyes, rare-earth emitters and quantum dots have been developed to boost the signal brightness and enhance the analytical performance of LFIAs [17,18]. However, quantum dots showed a dissatisfactory stability in an aqueous solution [19] and the aggregation-caused quenching (ACQ) phenomenon largely restricts the loading amount and fluorescent intensity of traditional fluorophores [20].

Encouragingly, since the first discovery of the aggregation-induced emission (AIE) effect by Tang and co-workers [21], AIE luminogens (AIEgens) have fundamentally overcome the ACQ limitations of traditional fluorophores [22,23,24,25,26]. In general, the radiative pathway of AIEgens is predominant for a bright emission via the restriction of intramolecular rotation, vibration and motion in the aggregated form, which is highly beneficial for increasing the fluorophore loading amount and improving the sensitivity of AIEgen nanoparticles (AIENPs). Consequently, AIENPs possess the superior merits of large absorptivity, robust luminosity and excellent photobleaching resistance. Therefore, they are a new generation of luminous labels that have appeared to improve the LFIA performance. However, the majority of current AIENPs emit short-wavelength fluorescence (<560 nm), which is difficult to circumvent the biological autofluorescence background interference. Notably, red-emissive AIENP labels are expected to solve this issue and increase the signal-to-noise ratio. To the best of our knowledge, a red-emissive AIENP-based LFIA has been barely reported.

**Scheme 1:**
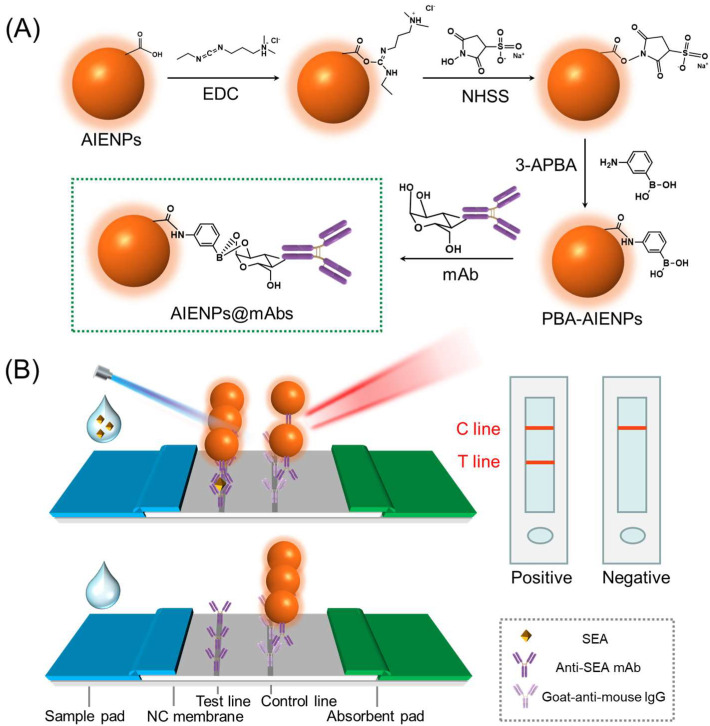
Schematic illustration of (**A**) fabrication of AIENPs@mAbs and (**B**) construction of sandwich PBA-AIENP-LFIA platform for SEA detection.

In this study, red-emissive AIENPs were introduced as the bright fluorescent label and their feasibility in a sandwich LFIA platform for the fast and sensitive detection of SEA in pasteurised milk was demonstrated (Scheme 1). The nanoprobe was first constructed by directly immobilising antibodies on boronate-tagged red-emissive AIENPs (PBA-AIENPs) via a boronate affinity reaction and it exhibited a high SEA-specific affinity and remarkable fluorescent performance. Various parameters of the PBA-AIENP-LFIA were systematically investigated to optimise the detection performance. Standard curves of the PBA-AIENP-LFIA for the quantitative identification of SEA in real pasteurised milk samples were constructed and the analytical performances, including the limit of detection (LOD), selectivity, accuracy, precision and versatility, were further systematically evaluated.

## 2. Results

### 2.1. Characterisation of AIENPs@mAbs

**Figure 1:**
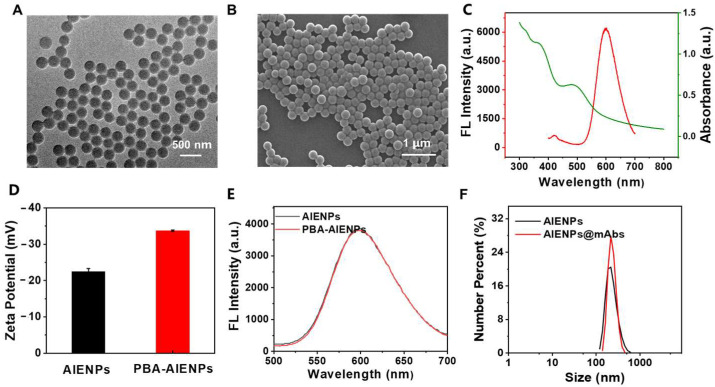
(**A**) TEM and (**B**) SEM images of AIENPs. (**C**) UV-vis absorption spectrum and fluorescence emission spectrum of AIENPs. (**D**) Zeta potential evolution and (**E**) fluorescence emission spectra of AIENPs and PBA-AIENPs. (**F**) Dynamic light scattering analysis of AIENPs and PBA-AIENPs@mAbs.

The typical transmission electron microscopy (TEM) image and SEM image in Figure 1A,B showed that the AIENPs presented a homogeneous spherical morphology and were well-dispersed, with a uniform size of 252.18 ± 16.75 nm (Figure S1). The dynamic light scattering analysis (Figure 1F) presented a hydrated diameter (D_H_) of 258.9 nm and a polydispersity index of ~0.1, indicating that the AIENPs possessed an excellent dispersity. The optical characteristics of the AIENPs were revealed by UV-vis absorption spectroscopy and fluorescence tests. As shown in Figure 1C, the AIENPs presented two absorption bands at 365 and 485 nm and displayed a bright red-emissive fluorescence signal at 598 nm under a 365 nm excitation. For the further construction of the anti-SEA mAb-labelled AIENPs, a boronate affinity reaction was chosen as an effective strategy due to the reduced antibody demand and superior antibody orientation according to previous studies [27]. Firstly, boric acid group-abundant surfaces on the AIENPs (PBA-AIENPs) were created by using the EDC technique to generate an amide connection between the carboxyl group of the AIENPs and the amino group of 3-APBA. The zeta potential analysis shown in Figure 1D indicated that the surface of the AIENPs was terminated by carboxyl groups; thus, it was negatively charged at −22.5 mV at a neutral pH. The modification of the PBA on the AIENPs adjusted the zeta potential to a more negative value of −33.5 mV, suggesting the successful modification of the boric acid groups on the AIENPs. In addition, the PBA-AIENPs maintained the fluorescent profile and brightness of the AIENPs (Figure 1E). Thereafter, by using a boronate affinity reaction between the glycosylated cis-diol on the Fc region of the anti-SEA mAbs and the boric acid group on the PBA-AIENPs, AIENPs@mAb conjugates were created. As shown in Figure 1F, the obtained AIENPs@mAbs exhibited an increased ∆D_H_ value of ~30 nm, which was well-matched with the spatial dimensions of the IgG molecule (14 nm × 8.5 nm × 4.5 nm) in a watery solution. This probably indicated that the anti-SEA mAbs were successfully conjugated on the surface of the PBA-AIENPs with an erected orientation. Meanwhile, a polydispersity index of less than 0.2 indicated that the AIENPs@mAb probes maintained an acceptable monodispersity.

### 2.2. Optimisation of AIENPs@mAb Immunoprobes

**Figure 2:**
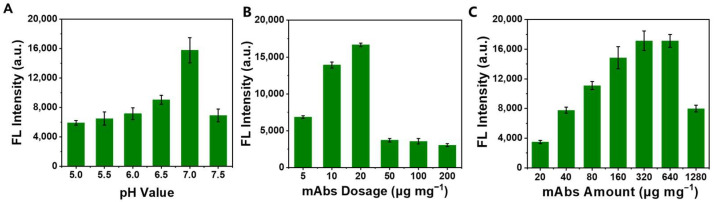
(**A**) Effect of pH on the coupling of anti-SEA mAbs and PBA-AIENPs. The added mAbs concentration was 20 µg mL^−1^. (**B**) The added amount of labelled anti-SEA mAbs on the coupling of anti-SEA mAbs and PBA-AIENPs at a pH of 7.0. (**C**) Effect of the added amount of labelled anti-SEA mAbs on the conjugation of anti-SEA mAbs and AIENPs by EDC method. The spiked SEA concentration was 20 ng mL^−1^.

The concept behind the developed PBA-AIENP-LFIA strip for sensitive SEA detection in pasteurised milk was based on a sandwich format, as presented in Scheme 1. When the SEA protein was present in the pasteurised milk, the antigens were firstly bound to the PBA-AIENPs@mAbs to form a PBA-AIENPs@mAb–SEA complex. After that, the intricate mixture was allowed to run on the LFIA test strip that had been made by applying anti-SEA mAbs to the NC membrane, resulting in the creation of the sandwich structure of the nanoprobe/antigen/antibody on the T line with brilliant fluorescent signals (FI_T_). Meanwhile, the goat-anti-mouse IgG caught extra PBA-AIENPs@mAbs on the C line with the accompanying fluorescent signal (FI_C_). The ratio of FI_T_/FI_C_ was used to accurately indicate the SEA concentration through circumventing the intensity fluctuation by the ratiometric strategy. First, anti-SEA mAbs were incubated with PBA-AIENPs at a pH of 5.0–7.5 in pasteurised milk in order to study the impact of the pH on the antibody coupling efficiency because the borate affinity efficacy was highly connected to the pH of the solution. As shown in Figure 2A, the optimal reaction pH appeared at 7.0 with the highest FI_T_ of 23,650; this was chosen as the working pH in the following assays. In addition, the mAbs amount modified on the particle surface substantially determined the immunoaffinity of the AIENPs@mAb nanoprobes and the sensitivity of the LFIA. Therefore, the modification of the optimal amount of anti-SEA mAbs was further assessed by tracking the FI_T_ change on the T line. As shown in Figure 2B, the FI_T_ values of the PBA-AIENPs@mAbs initially increased with the addition of mAbs and then reached the highest binding affinity of the PBA-AIENPs@mAb immunoprobes to SEA under an added mAbs amount of 20 µg per mg of PBA-AIENPs (2186 antibodies/NPs; 0.01 antibody/nm^2^). However, as the amount of added mAbs increased, the FI_T_ values fell, indicating an excess of immobilised antibodies attributed to antibody overcrowding and orientation deformation on the PBA-AIENP surface, thereby considerably decreasing the antigen accessibility of the PBA-AIENPs@mAbs [28,29,30]. In sharp contrast, the optimal mAbs coupling amount by the EDC method reached an extremely high level of 320 µg mg^−1^ with a comparative FI_T_ value (Figure 2C). The aforementioned findings supported the authors’ earlier report [27], showing that the borate affinity-mediated directional conjugation of mAbs (20 µg mg^−1^) could effectively reduce the added amount of antibodies with a high sandwich antibody–antigen combination efficiency. This was because the borate affinity could successfully preserve the biological activity of the antibody during the Fc-directed antibody conjugation. Therefore, the optimal anti-SEA mAbs amount was set as 20 µg mg^−1^ for further LFIA testing.

### 2.3. Optimisation of PBA-AIENP-LFIA Strip

Two key parameters, including the quantity of the captured antibody sprayed on the T line and the amount of PBA-AIENPs@mAbs, were carefully examined and optimised to attain the best SEA detection sensitivity. Figure S2 shows an optimised captured antibody level of 2 mg mL^−1^ because of the relatively high signal intensity and low antibody consumption. Meanwhile, as shown in Figure S3, with the increase in the added amount of immunoprobes, the C lines exhibited a conspicuous FI_C_ enhancement due to the specific combination of the immunoprobes and the goat-anti-mouse IgG. However, when SEA was absent, the FI_T_ underwent an obvious accompanying increase due to non-specific adsorption when the immunoprobe amount reached or exceeded 736 ng/strip. Therefore, the PBA-AIENPs@mAb nanoprobe amount was optimised as 368 ng/strip for further measurements [31].

### 2.4. Detection Performance of PBA-AIENP-LFIA for SEA Determination

**Figure 3:**
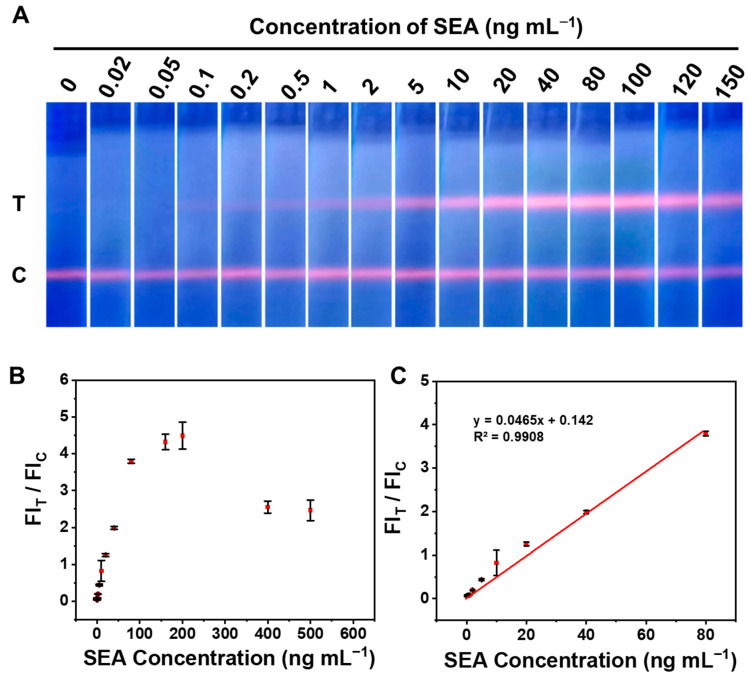
Quantitative detection of SEA via the proposed PBA-AIENP-LFIA platform. (**A**) Stereograms of PBA-AIENP-LFIA for SEA-spiked milk samples with SEA concentrations of 0, 0.02, 0.05, 0.1, 0.2, 0.5, 1, 2, 5, 10, 20, 40, 80, 100, 120 and 150 ng mL^−1^. (**B**) FI_T_/FI_C_ signal of the PBA-AIENP-LFIA platform at SEA concentrations ranging from 0 to 500 ng mL^−1^ in pasteurised milk. (**C**) Standard curve of PBA-AIENP-LFIA for SEA determination in pasteurised milk samples.

**Figure 4:**
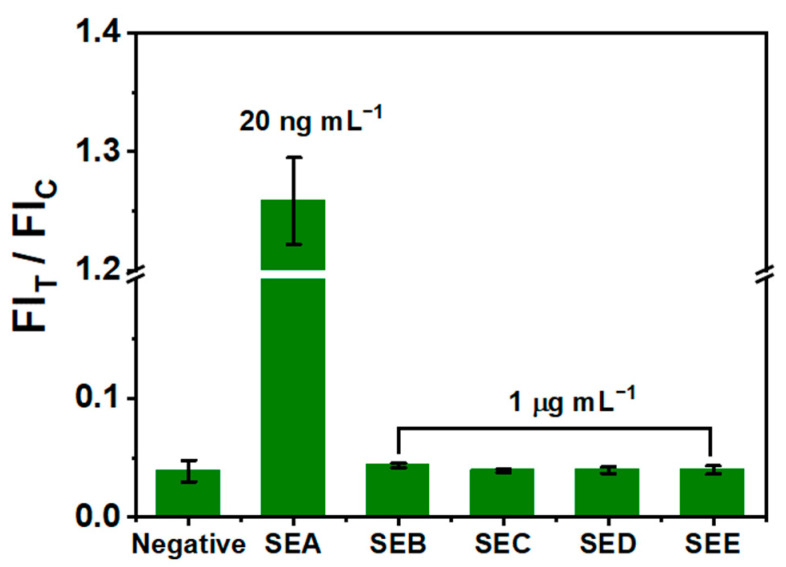
Evolution of the specificity of the PBA-AIENP-LFIA to staphylococcal enterotoxins (SEA, SEB, SEC, SED and SEE).

The designed PBA-AIENP-LFIA platform was used in the following experiments to sensitively detect SEA under the aforementioned ideal sensing circumstances. The stereogram of the strips showed that the FI_T_ increased with an increasing SEA concentration (Figure 3A). As shown in Figure 3B, the ratiometric FI_T_/FI_C_ value increased with the SEA concentration over the range of 0–200 ng mL^−1^ in milk, whereas the FI_T_/FI_C_ intensity decreased with an apparent ‘hook effect’ when the SEA concentration exceeded 200 ng mL^−1^. The calibrating curve for SEA in pasteurised milk (Figure 3C, Figure S4) demonstrated a linear correlation at SEA concentrations of 0–80 ng mL^−1^ with a linear regression equation of y = 0.0465 x + 0.142 (R^2^ = 0.9908), in which x and y are the SEA concentration and the FI_T_/FI_C_ intensity, respectively. The LOD of SEA in pasteurised milk was calculated as 0.04 ng mL^−1^, which was excellent compared with earlier established methods for SEA detection, as listed in Table S1. It also indicated the superiority of the fluorescent quantitative reading, with a better sensitivity than eye reading [32]. Another important issue for immunoassays is the detection specificity. In this study, SEB, SEC, SED and SEE staphylococcal enterotoxins were utilised as interferences to assess the selectivity of the PBA-AIENP-LFIA platform. The FI_T_/FI_C_ signals of SEA at a concentration of 20 ng mL^−1^ in pasteurised milk were compared with interference species at a concentration of 1 μg mL^−1^. As shown in Figure 4, a significant FI_T_/FI_C_ signal was produced with SEA, but the signals of the interfering species were almost undetectable. This result demonstrated that the proposed PBA-AIENP-LFIA platform could provide the target proteins with a strong specificity and distinguish SEA from complicated samples.

Moreover, the precision and accuracy of the PBA-AIENP-based LFIA were evaluated by identifying milk samples that had been tainted with SEA at doses of 5, 20 and 50 ng mL^−1^. Five replicates of each spiked concentration were run in the intra-assay over the course of one day, whereas the inter-assay was run continuously every day for three days. As presented in Table 1, the variance coefficients for all tests were less than 15% and the average recoveries varied from 91.3% to 117.6%. These results validated the reliability and practicality of the proposed PBA-AIENP-LFIA method for the quantitative detection of SEA in real milk samples.

SEA sensing in two other types of food matrix was also evaluated to further investigate the versatility of the proposed method. Figure S5A,B show the signal response and the calibration curve for SEA in chicken samples, demonstrating a linear correlation at SEA concentrations of 0.8–320 ng g^−1^ and a linear regression formula of y = 0.004x − 0.0034 (R^2^ = 0.995). Meanwhile, the calibration curve for SEA in salad samples demonstrated linear correlations at SEA concentrations of 0.8–640 ng g^−1^, with a linear relation of y = 0.004x − 0.0415 (R^2^ = 0.993; Figure S5C,D). The intra-assay and inter-assay results shown in Table 1 demonstrated average recoveries ranging from 84.4% to 122.8% and from 91.5% to 109.2% as well as variation coefficients of less than 15% for the chicken and salad samples, respectively. These results confirmed that the proposed PBA-AIENP-LFIA method possessed the potential for extensive applications in different food matrixes.

## 3. Conclusions

In summary, a novel red-emissive AIENP-based LFIA was successfully developed and first applied for SEA detection in multiple food matrixes. The AIENP fluorescent nanoprobe was synthesised by directly immobilising antibodies on boronate-tagged AIENPs through a boronate affinity reaction. It exhibited an excellent SEA-specific affinity and a remarkable fluorescent performance. The immunoreaction was systematically optimised and the ultrasensitive and fast detection of SEA in pasteurised milk was achieved within 20 min with LOD of 0.04 ng mL^−1^. In addition, the proposed PBA-AIENP-LFIA showed excellent specificity for the quantitative detection of SEA. The average recoveries of this PBA-AIENP-LFIA in pasteurised milk ranged from 91.3% to 117.6%, with a coefficient of variation below 15%. Moreover, the proposed method exhibited excellent feasibility in detecting SEA in chicken and salad samples, indicating its usefulness for a practical detection in different food matrixes. The developed PBA-AIENP-LFIA platform showed its potential as a strategic force for the identification of additional compounds in food samples as well as an ideal test method for field detection.

## 4. Materials and Methods

### 4.1. Materials

The red-emissive AIENPs were obtained from Weibang Biotechnology Co., Ltd. (Jiangxi, China). SEA, SEB, SEC, SED and SEE were bought from Beijing China Biotechnology Co., Ltd. (Beijing, China) 3-Aminophenylboronic hydrochloride (3-APBA), 1-ethyl-3-(3-dimethylaminopropyl) carbodiimide (EDC) and N-hydroxysulfosuccinimide sodium salt (NHSS) were purchased from Sigma–Aldrich (St. Louis, MI, USA) Chemical. Anti-SEA monoclonal antibodies (mAbs) were prepared through injecting SEA protein into BALB/c mice. The goat-anti-mouse IgG antibody was purchased from Thermo Fisher Scientific (Waltham, MA, USA). The sample pad, nitrocellulose (NC) membrane (with a pore size of ~15 µm and a wetting rate of 100 s/4 cm) and absorbent pad were obtained from Schleicher and Schuell GmbH (Feldbach, Switzerland). The phosphate buffer (PB, 0.01 M) was prepared by mixing 3.58 g of Na_2_HPO_4_∙12H_2_O and 1.56 g of NaH_2_PO_4_∙2H_2_O into 1 L of Milli-Q water and the pH was adjusted to 7.4 before use. All other reagents were purchased from Sinopharm Chemical Corp and were of an analytical grade and used without further purification.

### 4.2. Instruments and Characterisation

A JEM-1011 transmission electron microscope (TEM, 100 kV) was used to capture the TEM images. A Hitachi S-4800 scanning electron microscope (Tokyo, Japan) was used to take scanning electron microscopic (SEM) images by depositing 1 mg mL^−1^ of an AIENP aqueous dispersion onto a silicon wafer, followed by the sputtering of a gold layer before the observation. The hydrodynamic size and zeta potential were analysed using a scientific DLS NP analyser (Malvern Nano ZSE, London, UK) using 0.1 mg mL^−1^ of a PBA-AIENP aqueous dispersion with a 20 s equilibration time and 3 replicates at room temperature. The fluorescence spectra were obtained via a Hitachi F-4500 spectrophotometer. The motion controller, a BioJet Quanti3000k dispenser, and an AirJet Quanti3000k dispenser for solution dispensing were all included in the BioDot XYZ platform that was provided by BioDot (Irvine, CA, USA). We bought an automatic programmed cutter from Shanghai Jinbiao Biotechnology Co. (Shanghai, China). Fenghang Laboratory Instrument Co., Ltd. (Zhejiang, China) provided a portable fluorescent strip reader (Hangzhou, China).

### 4.3. Preparation of PBA-AIENPs

The EDC approach was used to create an amide bond between the carboxyl group of the AIENPs and the amino group of 3-APBA to prepare the PBA-AIENPs. Briefly, 1 mL of 15 mg mL^−1^ AIENPs were centrifuged at 15,640 g and redispersed in 0.5 mL of 0.01 M PB (pH of 6.0). A total of 0.25 mL of a PB (pH of 6.0) solution containing 8.9 mg EDC and 5.0 mg NHSS was then added into the AIENPs and the mixture was incubated with gentle stirring at room temperature for 2 h. After centrifuging and washing twice, 1 mL of the PB (pH of 8.5) solution containing 0.8 mg 3-APBA was added into the AIENPs for another 90 min reaction at room temperature and gently stirred. The obtained PBA-AIENPs were centrifuged and washed twice using 0.01 M PBS (pH of 7.0) containing 1 M NaCl and Milli-Q water to remove the non-specific adsorbed 3-APBA. Finally, they were redispersed in 1 mL Milli-Q water and stored at 4 °C for further use.

### 4.4. Preparation of AIENPs@mAbs

The AIENPs@mAb conjugates were prepared via the boronate affinity reaction to connect the boric acid group on the PBA-AIENPs and the carbohydrate residues on the Fc portion of the anti-SEA mAbs. In detail, in 1 mL of 0.01 M PB (pH of 7.4), 2 µL of 15 mg mL^−1^ PBA-AIENPs and 3.6 µL of 6 mg mL^−1^ anti-SEA mAbs were mixed. The mixture was then incubated at room temperature for 20 min whilst being gently stirred, followed by the addition of 100 µL casein (10% *w*/*v* in water) to react for another 30 min. The mixture was then centrifuged at 15,640 g for 15 min. The precipitates were redispersed in 60 µL of 0.01 M PB (pH of 7.4) that contained 25% *w*/*v* saccharose, 1% *w*/*v* BSA and 0.1% *w*/*v* sodium azide (NaN_3_). These were kept at 4 °C for use within a week.

In contrast, the AIENPs@mAbs generated by the EDC technique relied on the amido bond formation between the carboxyl group of the AIENPs and the amino group of the anti-SEA mAbs when EDC was present. In brief, 0.1 mg mL^−1^ of AIENPs and 3 μg of EDC were dissolved in 200 μL of 0.01 M PB (pH of 7.0), together with 6.4 μg of anti-SEA mAbs. The mixture was incubated for 90 min at room temperature and then blocked with BSA (5 mg) for another 60 min. The mixture was then centrifuged at 15,640 g for 15 min and the precipitates were then redispersed in 60 µL of 0.01 M PB (pH of 7.4) that contained 25% *w*/*v* saccharose, 1% *w*/*v* BSA and 0.1% *w*/*v* NaN_3_. These were kept at 4 °C for use within one week.

### 4.5. SEA Detection Using PBA-AIENP-LFIA Strip

The PBA-AIENP-LFIA strip was built according to our previous study [27]. First, 2 mg mL^−1^ of anti-SEA mAbs and 0.3 mg mL^−1^ of goat-anti-mouse IgG were sprayed on the NC membrane as the test (T) and control (C) lines, respectively. The modified NC membrane was then kept overnight at 37 °C to dry. Together with the absorbent pads, the treated NC membrane and sample pads were combined into test strips and then cut to a 3.9 mm width. The strips were put in a sealed bag and kept in a dry and cool environment for use for up to six months.

The target assay was carried out using the standard sandwich LFIA method as follows: 2 µL of the PBA-AIENPs@mAb probe was premixed with 70 µL of the sample solutions that were spiked with various SEA concentrations and incubated for 5 min at room temperature. The reacted mixture was then pipetted into the sample well. The strip was scanned using a commercial fluorescence strip reader after running for 15 min and the fluorescence intensities (FI) at the T and C lines (abbreviated as FI_T_ and FI_C_) were noted. By charting the linear relationship between FI_T_/FI_C_ and the target concentration, the standard curve for SEA was created. Three further measurements at each concentration were taken to obtain the error bars. The LOD was established using the mean concentration plus a three-fold standard deviation from twenty samples that tested negative for SEA.

### 4.6. Sample Preparation

Real milk as well as salad and chicken samples, confirmed to be without SEA by electrophoretic and immunoblot analyses in accordance with the industrial standard (SN/T 2416-2010 (China)), were acquired at a neighbourhood supermarket in Nanchang, China. Briefly, the sample was prepared in the manner described below. For the chicken and salad, 10 g of the samples was mixed with 40 mL water and underwent homogeneous processing. The supernatant was kept at −20 °C for later use after centrifugation at 8000 g for 20 min. For the milk, the samples were directly spiked with SEA for the PBA-AIENP-LFIA strip detection.

## Figures and Tables

**Table 1:** Precision and accuracy of PBA-AIENP-based LFIA test strips for detection of SEA in pasteurised milk as well as chicken and salad samples.

Sample	SEA (ng mL^−1^)	Intra-Assay ^a^			Inter-Assay ^b^		
		Mean	Recovery (%)	CV (%)	Mean	Recovery (%)	CV (%)
Pasteurised Milk	5	4.9	99.2	6.2	5.72	114.4	6.8
	20	17.7	91.3	11.8	19.3	96.2	5.9
	50	59.8	117.6	8.7	55.6	111.2	7.4
Chicken	5	4.3	85.1	6.4	6.14	122.8	8.6
	20	17.8	89.1	2.5	17.2	86.0	5.5
	50	45.2	90.4	10.2	42.2	84.4	12.5
Salad	5	5.4	108.0	4.4	5.46	109.2	7.6
	20	19.6	98.0	8.6	18.4	91.9	8.0
	50	48.4	96.8	4.7	45.8	91.5	2.9

^a^ Intra-assay was obtained from five replicates at each concentration. ^b^ Inter-assay was conducted once daily for 3 days continuously.

## Data Availability

Not applicable.

## Supplementary Materials

The following supporting information can be downloaded at https://www.mdpi.com/article/10.3390/toxins15020113/s1: Figure S1: Optimisation of the sprayed amount of anti-SEA mAbs onto the T line; Figure S2: Effect of PBA-AIENPs@mAb immunoprobe amount on the FI_T_ and FI_C_ of the PBA-AIENP-LFIA test strips; Figure S3: Standard curve of PBA-AIENP-LFIA for SEA determination in chicken and salad samples. Figure S4: Fluorescent signal change of PBA-AIENPs-LFIA in responding to logarithmic SEA concentration in pasteurised milk in the range of 0–80 ng mL^−1^. Figure S5: Fluorescent signal change in responding to logarithmic concentration and standard curve of PBA-AIENPs-LFIA for SEA determination in (A,B) chicken and (C,D) salad samples in the range of 0.8–320 ng g^−1^ and 0.8–640 ng g^−1^, respectively. Table S1: Comparison of the analytical performances of the proposed method with the reported methods for SEA detection. References [33,34,35,36,37,38,39,40,41,42,43,44,45,46,47,48,49,50,51] are cited in the supplementary materials.
