# Supplementary material for: Aggregation-Induced Red Emission Nanoparticle-Based Lateral Flow Immunoassay for Highly Sensitive Detection of Staphylococcal Enterotoxin A
Source: Toxins (Basel). 2023 Jan 29;15(2):113. doi: 10.3390/toxins15020113 (PMC9964500; doi:10.3390/toxins15020113)
Supplement: Supplementary file 1 [file toxins-15-00113-s001.zip › toxins-2141447-supplementary.pdf]

Supplementary Materials: Aggregation-Induced Red Emission Nanoparticle-Based Lateral Flow Immunoassay for Highly Sensitive Detection of Staphylococcal Enterotoxin A

Hanpeng Xiong, Ping Chen, Xirui Chen, Xuanang Shen, Xiaolin Huang, Yonghua Xiong and Yu Su

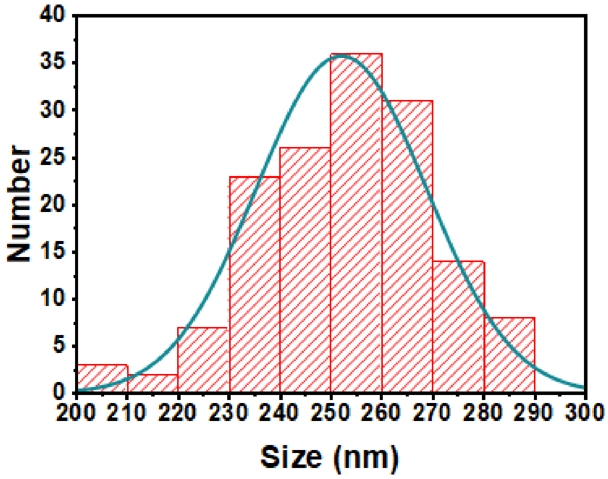

Figure S1. Size statistic of the AIENPs by measuring 150 particles in the SEM image in **Figure 1**B using Image J.

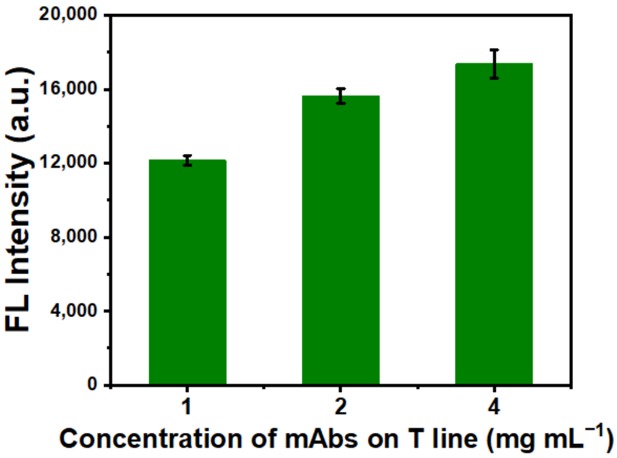

Figure S2. Optimization of the sprayed amount of anti-SEA mAbs onto the T line with a spiked SEA concentration of 20 ng mL^{-1}.

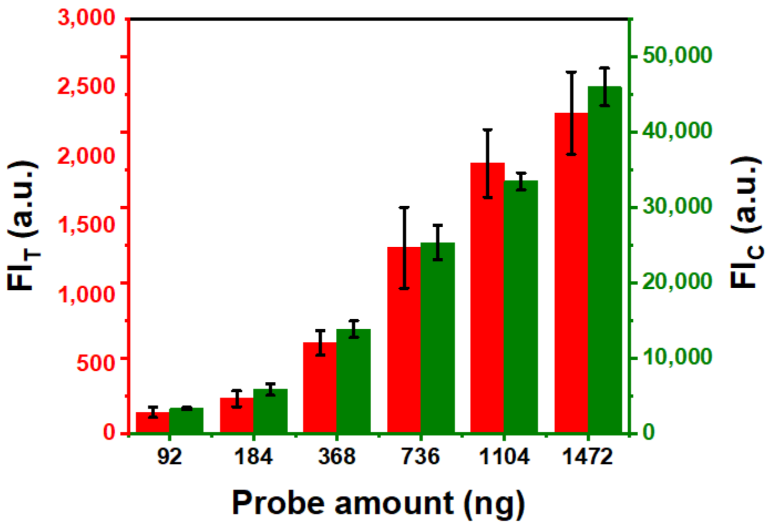

Figure S3. Effect of PBA-AIENPs@mAbs immunoprobes amount on the FI_T and FI_C of the PBA-AIENPs-LFIA test strips with a T line mAbs concentration of 2 mg mL^{-1} and C line goat anti-mouse IgG concentration of 0.3 mg mL^{-1}.

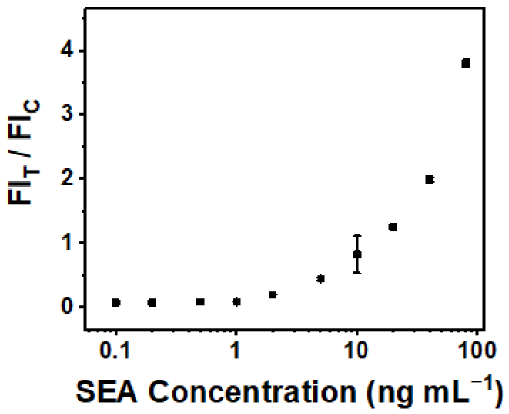

Figure S4. Fluorescent signal change of PBA-AIENPs-LFIA in responding to logarithmic SEA concentration in pasteurised milk in the range of 0-80 ng mL^{-1}.

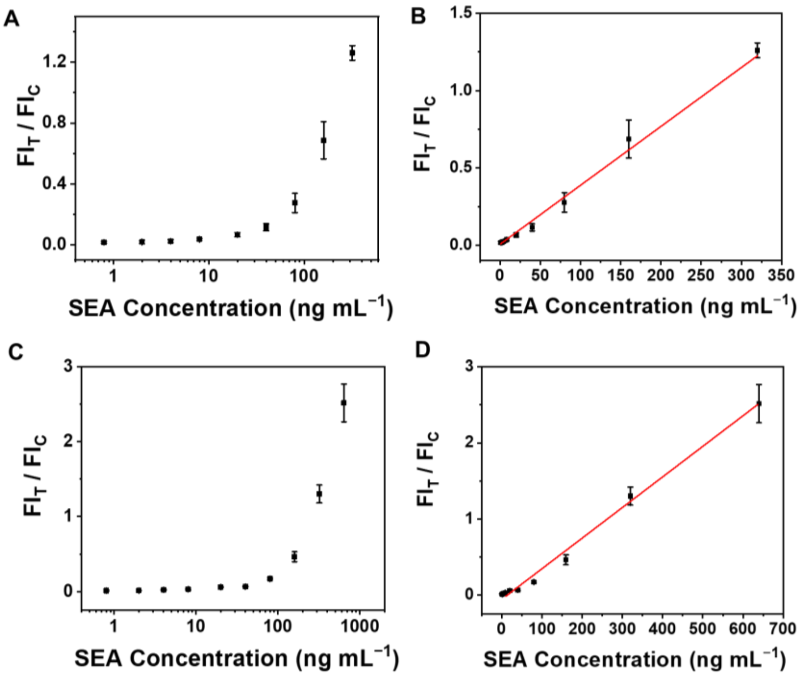

Figure S5. Fluorescent signal change in responding to logarithmic concentration and standard curve of PBA-AIENPs-LFIA for SEA determination in (A,B) chicken and (C,D) salad samples in the range of 0.8-320 ng g^{-1} and 0.8-640 ng g^{-1}, respectively.

Table S1. Comparison of the analytical performances of the proposed method with the reported methods for SEA detection.

Method	Labels	LOD (ng mL^{-1})	Linear Range (ng mL^{-1})	Sample matrix	Sample volume (μL)	Time (min)	Reference
Colorimetry	/	0.0282	0.06–2	Milk	/	/	[1]
Colorimetry	Antibodies	0.064	0–0.5	Milk	/	/	[2]
Colorimetry	AuNPs	2.5	5–100	Milk	/	15	[3]
Colorimetry	HRP-antibodies	1	0–100	Milk	100	/	[4]
Fluorescent	MNPs	0.23	0.25–5	Milk	200	30	[5]
Fluorescent	DNA-AgNCs	0.3393	0.5–1000	Milk	100	30	[6]
Fluorescent	QBs	1.89	2–150	Milk	/	15	[7]
Fluorescent	Fluorescent SEA	0.0045	0.45–9.75	Water	/	/	[8]
Fluorescent	MNPs and CQDs	0.002	0.004–2	Milk	100	/	[9]
QCM	Polypyrrole	0.4	1–80	Chicken and Milk	/	30	[10]
QCM	/	7	50–1000	PBS	/	25	[11]
QCM	AuNPs	1	50–500	Water	/	25	[12]
LC-MS/MS	/	8	/	Milk and buffer	1000	/	[13]
BIA-MS	/	1	/	Milk and mushroom	/	/	[14]
LSPR	AuNP-Abs	0.5	10–250	Milk	300	/	[15]
PCR	Phage	0.1	1–10	Milk	/	/	[16]
Electrochemical	Gold electrode	33.9	16–150	Cheese	/	/	[17]
Flow Cytometry	Functional beads	0.25	0.02–20	Liquid samples	/	/	[18]
SPR	Thiol-coated gold disks	100	100–1000	Milk	/	/	[19]
Fluorescent	AIENPs	0.04	0–80	Milk and chicken salad	/	20	This study

Reference

1. Kuang, H.; Wang, W.; Xu, L.; Ma, W.; Liu, L.; Wang, L.; Xu, C., Monoclonal antibody-based sandwich ELISA for the detection of staphylococcal enterotoxin A. *International Journal of Environmental Research and Public Health* **2013,** *10* (4), 1598-1608.
2. Clarisse, T.; Michèle, S.; Olivier, T.; Valérie, E.; Jacques-Antoine, H.; Michel, G.; Florence, V., Detection and quantification of staphylococcal enterotoxin A in foods with specific and sensitive polyclonal antibodies. *Food Control* **2013,** *32* (1), 255-261.
3. Wang, W.; Liu, L.; Xu, L.; Kuang, H.; Zhu, J.; Xu, C., Gold-nanoparticle-based multiplexed immunochromatographic strip for simultaneous detection of staphylococcal enterotoxin A, B, C, D, and E. *Particle & Particle Systems Characterization* **2016,** *33* (7), 388-395.
4. Freed, R. C.; Evenson, M. L.; Reiser, R. F.; Bergdoll, M. S., Enzyme-linked immunosorbent assay for detection of staphylococcal enterotoxins in foods. *Applied and Environmental Microbiology* **1982,** *44* (6), 1349-1355.
5. Becheva, Z.; Ivanov, Y.; Gabrovska, K.; Godjevargova, T., Rapid immunofluorescence assay for staphylococcal enterotoxin A using magnetic nanoparticles. *International Journal of Food Science & Technology* **2019,** *54* (3), 916-922.
6. Zhang, X.; Khan, I. M.; Ji, H.; Wang, Z.; Tian, H.; Cao, W.; Mi, W., A label-free fluorescent aptasensor for detection of staphylococcal enterotoxin A based on aptamer-functionalized silver nanoclusters. *Polymers* **2020,** *12* (1), 152.
7. Chen, P.; Zhou, M.; Chen, X.; Xiong, S.; Su, Y.; Zhou, H.; Peng, J.; Xiong, Y., Quantum dot bead-based competitive immunochromatographic assay for enterotoxin aureus A detection in pasteurized milk. *Journal of Dairy Science* **2022,** *105* (6), 4938-4945.
8. Lam, M. T.; Wan, Q.; Boulet, C. A.; Le, X. C., Competitive immunoassay for staphylococcal enterotoxin A using capillary electrophoresis with laser-induced fluorescence detection. *Journal of Chromatography A* **1999,** *853* (1-2), 545-553.
9. Veissi, M.; Maktabi, S.; Ramezani, Z.; Khosravi, M., Highly Sensitive Fluorescence Assay of Enterotoxin A in Milk Using Carbon Quantum Dots as a Fluorophore. *Food Analytical Methods* **2021,** *14* (9), 1815-1825.
10. Karaseva, N.; Ermolaeva, T., A regenerable piezoelectric immunosensor on the basis of electropolymerized polypyrrole for highly selective detection of Staphylococcal Enterotoxin A in foodstuffs. *Microchimica Acta* **2015,** *182* (7), 1329-1335.
11. Salmain, M.; Ghasemi, M.; Boujday, S.; Spadavecchia, J.; Técher, C.; Val, F.; Le Moigne, V.; Gautier, M.; Briandet, R.; Pradier, C.-M., Piezoelectric immunosensor for direct and rapid detection of staphylococcal enterotoxin A (SEA) at the ng level. *Biosensors and Bioelectronics* **2011,** *29* (1), 140-144
12. Haddada, M. B.; Salmain, M.; Boujday, S., Gold colloid-nanostructured surfaces for enhanced piezoelectric immunosensing of staphylococcal enterotoxin A. *Sensors and Actuators B: Chemical* **2018,** *255*, 1604-1613.
13. Andjelkovic, M.; Tsilia, V.; Rajkovic, A.; De Cremer, K.; Van Loco, J., Application of LC-MS/MS MRM to determine staphylococcal enterotoxins (SEB and SEA) in milk. *Toxins* **2016,** *8* (4), 118.
14. Nedelkov, D.; Rasooly, A.; Nelson, R. W., Multitoxin biosensor–mass spectrometry analysis: a new approach for rapid, real-time, sensitive analysis of staphylococcal toxins in food. *International journal of food microbiology* **2000,** *60* (1), 1-13.
15. Ben Haddada, M.; Hu, D.; Salmain, M.; Zhang, L.; Peng, C.; Wang, Y.; Liedberg, B.; Boujday, S., Gold nanoparticle-based localized surface plasmon immunosensor for staphylococcal enterotoxin A (SEA) detection. *Analytical and bioanalytical chemistry* **2017,** *409* (26), 6227-6234.
16. Artykov, A.; Fursova, K.; Ryazantsev, D. Y.; Shchannikova, M.; Loskutova, I.; Shepelyakovskaya, A.; Laman, A.; Zavriev, S.; Brovko, F., Detection of staphylococcal enterotoxin a by phage display mediated immuno-PCR method. *Russian Journal of Bioorganic Chemistry* **2017,** *43* (5), 540-543.
17. Pimenta-Martins, M. G. R.; Furtado, R. F.; Heneine, L. G. D.; Dias, R. S.; de Fátima Borges, M.; Alves, C. R., Development of an amperometric immunosensor for detection of staphylococcal enterotoxin type A in cheese. *Journal of Microbiological Methods* **2012,** *91* (1), 138-143.
18. Shepelyakovskaya, A.; Rudenko, N.; Karatovskaya, A.; Shchannikova, M.; Shulcheva, I.; Fursova, K.; Zamyatina, A.; Boziev, K.; Oleinikov, V.; Brovko, F., Development of a bead-based multiplex assay for the simultaneous quantification of three staphylococcal enterotoxins in food by flow cytometry. *Food Analytical Methods* **2020,** *13* (5), 1202-1210.
19. Tsai, W.-C.; Li, I.-C., SPR-based immunosensor for determining staphylococcal enterotoxin A. *Sensors and Actuators B: Chemical* **2009,** *136* (1), 8-12.

Disclaimer/Publisher's Note: The statements, opinions and data contained in all publications are solely those of the individual author(s) and contributor(s) and not of MDPI and/or the editor(s). MDPI and/or the editor(s) disclaim responsibility for any injury to people or property resulting from any ideas, methods, instructions or products referred to in the content.
